# SERS Sensing Properties of New Graphene/Gold Nanocomposite

**DOI:** 10.3390/nano9091236

**Published:** 2019-08-30

**Authors:** Giulia Neri, Enza Fazio, Placido Giuseppe Mineo, Angela Scala, Anna Piperno

**Affiliations:** 1 Department of Chemical, Biological, Pharmaceutical and Environmental Sciences, University of Messina, Viale F. Stagno D’Alcontres 31, I-98166 Messina, Italy; 2 Department of Mathematical and Computational Sciences, Physics Science and Earth Science, University of Messina, Viale F. Stagno D’Alcontres 31, I-98166 Messina, Italy; 3 Department of Chemical Sciences, University of Catania, V.le A. Doria 6, 95125 Catania, Italy; 4 Institute for Chemical and Physical Processes-National Research Council (IPCF-CNR), Viale F. Stagno d’Alcontres 37, I-98158 Messina, Italy

**Keywords:** graphene/gold nanocomposite, SERS, Dopamine, Rhodamine 6G

## Abstract

The development of graphene (G) substrates without damage on the sp^2^ network allows to tune the interactions with plasmonic noble metal surfaces to finally enhance surface enhanced Raman spectroscopy (SERS) effect. Here, we describe a new graphene/gold nanocomposite obtained by loading gold nanoparticles (Au NPs), produced by pulsed laser ablation in liquids (PLAL), on a new nitrogen-doped graphene platform (G-NH_2_). The graphene platform was synthesized by direct delamination and chemical functionalization of graphite flakes with 4-methyl-2-*p*-nitrophenyl oxazolone, followed by reduction of *p*-nitrophenyl groups. Finally, the G-NH_2_/Au SERS platform was prepared by using the conventional aerography spraying technique. SERS properties of G-NH_2_/Au were tested using Rhodamine 6G (Rh6G) and Dopamine (DA) as molecular probes. Raman features of Rh6G and DA are still detectable for concentration values down to 1 × 10^−5^ M and 1 × 10^−6^ M respectively.

## 1. Introduction

Since their discovery, graphene materials (G), due to their outstanding physicochemical properties [1], have generated huge interest in numerous fields including biomedicine, electronics, sensing, energy, etc. [2,3,4,5,6,7,8]. They have been proposed as drug delivery systems for photothermal [9] and photodynamic therapy [10], as scaffold in tissue engineering [11], and as materials for biosensing [12,13]. Recently, G and its functionalized derivatives have been investigated as substrates for SERS (surface enhanced Raman spectroscopy) applications [14,15], a versatile technique that enables the rapid detection of various types of molecules [16,17].

Metal nanoparticles (i.e., Cu, Ag, gold nanoparticles (Au NPs)) are the most extensively studied SERS-active substrates since their collective electronic excitations, namely surface plasmons, are very interesting for a large variety of applications. Localized surface plasmon resonance excitation in Ag and Au NPs produces strong extinction and scattering spectra, resulting in amplification of the electric field (E) near the particle surfaces such that |E|^2^ can be 100–10,000 times greater in intensity than the incident field, which acts on a spatial range of 10–50 nm. These effects are mainly influenced by two factors: (i) NPs morphology (in terms of size and shape) and (ii) local dielectric environment [18,19,20].

Two main mechanisms are involved in Raman signal enhancement: The electromagnetic mechanism (EM), due to the strong amplification of the local EM field [21], and the chemical effect (CM) that involves the creation of new electronic states generated by the interaction between the metal and the molecules adsorbed on it [22]. Such new electronic states allow for resonant Raman scattering processes; the control of the distances among the localized surface plasmons, on a sub-nanometer scale, is a critical parameter to control the inter-particle optical coupling and therefore, the efficiency of SERS response [23].

Recently, engineered G have attracted a huge amount of attention as platforms for biological SERS sensing [24]. G offer a large flat surface to adsorb molecules through π–π interactions determining the manner in which molecules bind with the surface which, in turn, determines the symmetry of the molecules and the effective charge transfer [25]. However, G alone provides a limited enhancement factor [26] while the combination of specifically designed G with metallic NPs is an interesting strategy to obtain new materials with synergistic effect and improved SERS sensing performance. The high compatibility of G with metal noble NPs is mainly due to: (i) Transparency to laser light and localized plasmonic fields; (ii) high thermal conductivity; and (iii) appropriate dielectric strength that confines the plasmonic field [27,28].

**Figure 1:**
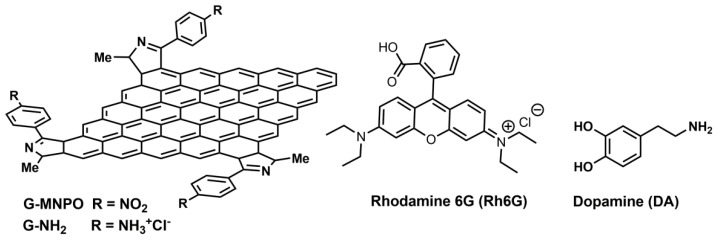
Schematic representation of G-MNPO and G-NH_2_. Chemical structure of Rhodamine 6G (Rh6G) and Dopamine (DA).

Despite the potentiality of these hybrid systems, the critical point of G-based SERS substrates regards the development of chemical strategies avoiding the damage of the G sp^2^ network, keeping an electron high mobility and, at the same time, enabling the tuning of the interactions with plasmonic surface to finally enhance SERS effects. Generally, 2D materials were obtained by liquid chemical exfoliation of related 3D stratified bulk materials, processes that required the presence of intercalation agents and ultrasonication treatment. To overcome the long processing times and to guarantee the quality of 2D substrates, the exfoliation methods have been continuously implemented [29,30]. Recently, we have developed a straightforward method for the direct delamination of graphite flakes into functionalized G with preserved sp^2^ network [31]. G-MNPO platform (Figure 1) [31], obtained by solvent-free 1,3-dipolar cycloaddition reaction of 4-methyl-2-*p*-nitrophenyl oxazolone with graphite, was selected for the development of a new nitrogen-doped graphene network (G-NH_2_). The amine groups, obtained by reduction of *p*-nitrophenyl group on the ∆-1-pyrrolidine rings, were envisaged as anchoring sites for Au NPs. Here, we report the synthesis and characterization of graphene/gold nanocomposite (G-NH_2_/Au) obtained by mixing G-NH_2_ and Au NPs. Au NPs were produced by pulsed laser ablation in liquids (PLAL) technique that allowed the production of metal NPs in a variety of solvents with tuned size and optical properties [32,33]. No surfactant is needed to stabilize the colloids obtained by PLAL, and the NPs are extremely pure without any post-synthesis treatment [31]. To the best of our knowledge, no data have been reported in the literature about the SERS properties of G/Au platforms, where Au NPs were produced by PLAL technique.

The chemical composition and the morphology of G-NH_2_ and G-NH_2_/Au platforms were investigated by micro-Raman and X-ray photoelectron (XPS) spectroscopies, scanning transmission electron microscopy (STEM), and thermogravimetric analysis (TGA).

The G-NH_2_/Au dispersion was transferred onto a glass slide to obtain a uniform nanostructure thick film and its SERS properties were tested using Rhodamine 6G (Rh6G) and Dopamine (DA) as molecular probes (Figure 1).

## 2. Materials and Methods

### 2.1. Materials

Graphite flakes, Dopamine, Rhodamine 6G, solvents, and other reagents were purchased from Sigma Aldrich, (Milan, Italy); gold target (high purity, 99.99%) was purchased from Mateck srl (Jülich, Germany).

### 2.2. Synthesis of G-NH_2_

G-MNPO was prepared according to the synthetic method already reported [31]. Further, 240 mg of G-MNPO, (0.09 mmol of NO_2_) were homogenously dispersed in 30 mL H_2_O by sonication (30 min). NaBH_4_ (100 mg, 2.63 mmol) was added and the reaction mixture was stirred at 80 °C for 12 h. Afterwards, the reaction mixture was cooled to room temperature (r.t.), acidified to pH 3 by addition of a HCl 1M solution, and stirred for 1 h at r.t. G-NH_2_ was recovered by filtration under vacuum (Millipore 0.1 µm) and it was purified by washing with 1:1 water/ethanol mixture. Finally, the residue was dried at ~60 °C to recover 185 mg of G-NH_2_.

### 2.3. Synthesis of Au NPs by PLAL

Au water colloids were prepared according to previously reported procedure [34] using the 532 nm second harmonic emission wavelength of a Nd:YAG laser (Tempest- Laser Point srl, Milan Italy) operating at a repetition rate of 10 Hz (pulse length: 5 ns).

### 2.4. Synthesis of G-NH_2_/Au

First, 20 mL of Au NPs were added to a dispersion of G-NH_2_ (52 mg) in water (2 mL), obtained by sonication for 10 min, and the mixture was ultrasonicated (65% W) for 30 min. The reaction mixture was filtered at reduced pressure (Millipore 0.1 µm), the solid was repeatedly washed with water, and after drying at ~60 °C, 44 mg of G-NH_2_/Au were recovered.

### 2.5. Preparation of G-NH_2_/Au SERS Platform

The aqueous dispersion of G-NH_2_/Au (5 mg/mL) was deposited onto a glass slide using the conventional aerography spraying technique. The aerography spraying system is made up by a high-pressure air brush with interchangeable nozzles of different sizes. During the deposition, the nozzle is continuously moved to ensure a uniform distribution on the substrate. The spraying is carried out in a deposition chamber equipped with a heated substrate holder and an excess vapors removal system to guarantee standard and reproducible conditions. The GNH_2_/Au SERS platform was tested for Rhodamine 6G (Rh6G) at concentrations of 1 × 10^−3^, 2 × 10^−4^, 5 × 10^−5^ M and for Dopamine (DA) at concentrations of 1 × 10^−3^, 2 × 10^−4^, 5 × 10^−5^, and 5 × 10^−6^ M. Rh6G and DA solutions were prepared using deionized water. The excitation sources were the 532 nm and 638 nm diode laser lines. The substrates were dipped in these solutions for 30 min and then taken out for free drying, after which the surface enhanced Raman (SERS) spectra were collected.

### 2.6. Samples Characterization

Thermal gravimetric analysis (TGA) profiles were acquired Perkin-Elmer Pyris TGA7 in the temperature range of 50–1000 °C. G-NH_2_ or G-NH_2_/Au (about 5 mg) were placed in a platinum pan and kept at 25 °C under a 60 mL min^−1^ air flow until balance stabilization (balance sensitivity was 0.01 mg), and subsequently heated with a scan rate of 10 °C min^−1^ under the same air flux. The calibration of instrument was settled according to previously reported procedure [31].

X-ray photoelectron spectroscopy was used to determine the surface elemental composition of the material and their bonding configurations. The spectra were acquired using a K-Alpha system (Thermo-Scientific, Germany) equipped with a monochromatic Al-Kα source (1486.6 eV), and operating in constant analyzer energy mode (pass energy: 200 eV), according to previously reported protocol [35]. Samples (G-NH_2_, G-NH_2_/Au, AuNPs) were deposited on a nickel grid to carry out scanning transmission electron microscopy (STEM) using a ZEISS instrument Merlin-Gemini 2 column (Merlin-Gemini, Germany), operating at primary voltage of 30 kV and at the working distance of 4 mm.

Raman spectra were acquired using the Horiba XploRA spectrometer (HORIBA Instruments, Milan, Italy) coupled with an optical microscope equipped with the 50X and 100X objectives. The excitation wavelengths used were 532 nm and 638 nm coming from solid diode lasers. The integration time was varied from 5 to 120 s, with an accumulation time of 2s, in order to optimize the signal to noise.

UV-vis optical absorption spectra of the Au and G-NH_2_/Au samples were recorded using quartz cells and a Perkin Elmer (Lambda 750 model) spectrometer (Perkin Elmer, Milan, Italy) working in the 300–900 nm range.

## 3. Results

### 3.1. G-NH_2_/Au SERS Platform

**Figure 2:**
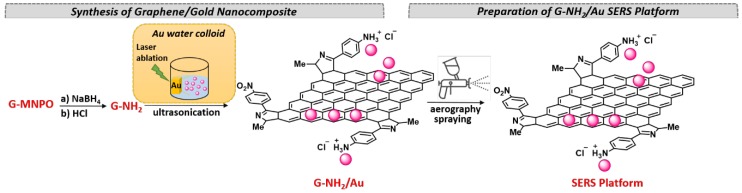
Preparation of G-NH_2_/Au surface enhanced Raman spectroscopy (SERS) platform.

SERS platform based on graphene/gold nanocomposite (G-NH_2_/Au) was obtained through a procedure involving: (i) Synthesis of G-NH_2_ by reduction of G-MNPO; (ii) Preparation of G-NH_2_/Au and (iii) Deposition of G-NH_2_/Au onto a glass slide by an aerography spraying probe (Figure 2).

G-MNPO was prepared according to the synthetic method previously reported [31], carrying out the cycloaddition reaction at the molar ratio of 1:7 flake graphite/oxazolone. The experimental conditions for G-MNPO synthesis were optimized to obtain a substrate with a large surface area and a high degree of functionalization (0.037 mmol of NO_2_/100 mg). G-MNPO was reduced with NaBH_4_ and converted in the protonated salt by treatment with hydrochloric acid. The cationic centers on G-NH_2_ surfaces increased the G dispersibility in water and guaranteed a better interaction with Au NPs. G-NH_2_/Au nanocomposite was obtained by mixing, under ultrasonication treatment, the aqueous dispersion of G-NH_2_ with the freshly prepared colloidal dispersion of Au NPs [34]. Finally, the aqueous dispersion of G-NH_2_/Au was deposited onto the glass slide.

### 3.2. Characterization of Graphene/Gold Nanocomposite (G-NH_2_/Au)

**Figure 3:**
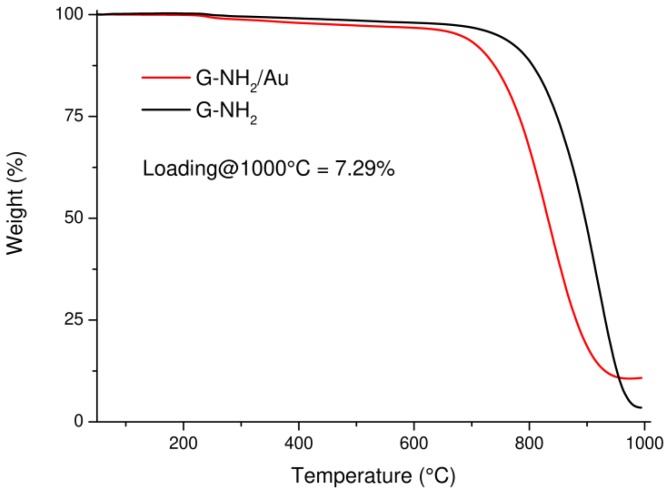
TGA profiles of G-NH_2_ and G-NH_2_/Au under air atmosphere.

The content of Au NPs on G was estimated by TGA under air atmosphere (Figure 3). TGA profiles of G-NH_2_ and G-NH_2_/Au showed a high thermal stability without significant weight loss under 600 °C, indicating the absence of labile oxygen-containing functional groups. TGA profile of G-NH_2_ showed a decomposition between 750 °C and 900 °C, with a complete decomposition of carbon at temperatures higher than 900 °C; whereas the G-NH_2_/Au profile showed a lower decomposition temperature between 600 °C and 800 °C and the decomposition of carbon content became remarkable at 800 °C. The lower thermal decomposition of G-NH_2_/Au compared to G-NH_2_ could be attributed to the presence of Au NPs that increased the interlayer spacing and porosity of G-NH_2_/Au. The residual mass of 7.29% indicated the loading of Au NPs on G-NH_2_/Au nanocomposite.

**Figure 4:**
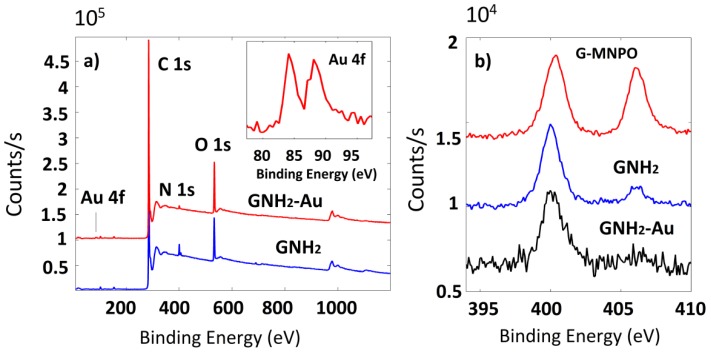
(**a**): XPS wide scan of G-NH_2_ and G-NH_2_/Au samples and line shapes of Au 4f (inset). (**b**): N 1s photoelectron deconvoluted line shapes of G-MNPO, G-NH_2_, and G-NH_2_/Au.

Detailed information about the functionalities on G surfaces were obtained by XPS analysis. The wide scan spectra of G-NH_2_ and G-NH_2_/Au were reported in Figure 4a, with the Au 4f profile in the inset. This profile was characterized by well-separated spin-orbit components (Δ = 3.7 eV) where the Au 4f peak was centered at the binding energy of 84.0 eV, which is characteristic of the metal Au species. The Au, C, O, and N relative atomic percentages are reported in Table 1. The Au weight content percentage calculated by XPS was in good agreement with TGA data (Table 1). The N 1s high-resolution profile of G-NH_2_ (Figure 4b) showed the presence of two peaks centered at about 400 eV, attributed to N=C and –NH_3_^+^ species, and at 407 eV due to NO_2_. The lower contribution of the peak at 407 eV in G-NH_2_ sample, compared with G-MNPO (20.05% vs. 41.18%, see Figure 4b and Table 1), indicated a good reduction of nitro groups into amino groups. The decrease of the oxygen content after the reduction reaction (19% vs. 7.4%, see Table 1) was connected with the changes observed by N 1s profile.

**Figure 5:**
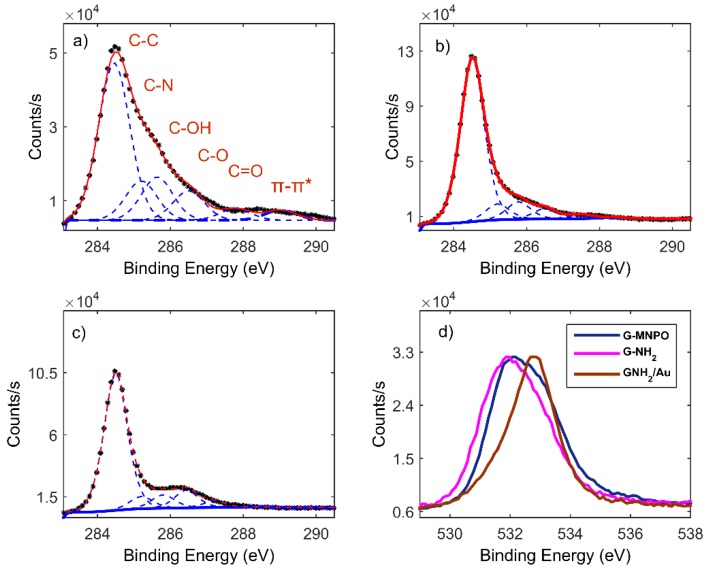
C 1s photoelectron deconvoluted line shapes: (**a**) G-MNPO, (**b**) G-NH_2_, (**c**) G-NH_2_/Au. (**d**) O 1s photoelectron deconvoluted line shapes of G-MNPO (blue), G-NH_2_ (violet), G-NH_2_/Au (brown).

C 1s profiles of G-MNPO, G-NH_2_, and G-NH_2_/Au were deconvolved considering six spectral components: A main contribution at 284.5 eV attributed to C=C/C–C in the aromatic ring, and four other contributions, at higher binding energies, corresponding to carbon atoms bonded to nitrogen (C–N) and oxygen (C–OH, C–O, C=O) centered at 285.2, 286.3, 288.7, and 288.9 eV, respectively. The contribution at about 291.0 eV referred to π–π* bonds (Figure 5).

**Figure 6:**
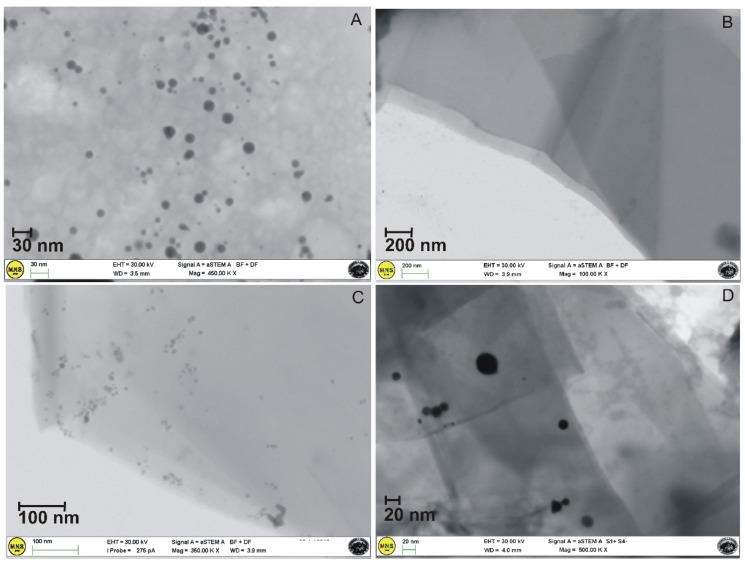
STEM images. (**A**) Au NPs produced by the green pulsed laser ablation technique in water at the laser fluence F of 1.5 J/cm^2^ and the irradiation time t of 20 min. The NPs are nearly spherical in shape with a mean diameter of 15 nm; (**B**) G-NH_2_ network with exfoliated G layers and transparent sheets, stacked onto each other, with a thickness of about 2–3 nm; (**C**,**D**) G-NH_2_/Au platform, with Au NPs embedded within the overlapped thin layers of graphene.

Morphological information about the size and distribution of Au NPs within the G layers was obtained by electron microscopy analyses. STEM images (Figure 6) showed homogeneously distributed exfoliated G layers and various dimensional transparent sheets, in several portions of the sample, stacked onto each other, with a thickness of about 2–3 nm. Moreover, Au NPs characterized by an average size of 15 nm were mainly distributed at the edges of the G layers.

**Figure 7:**
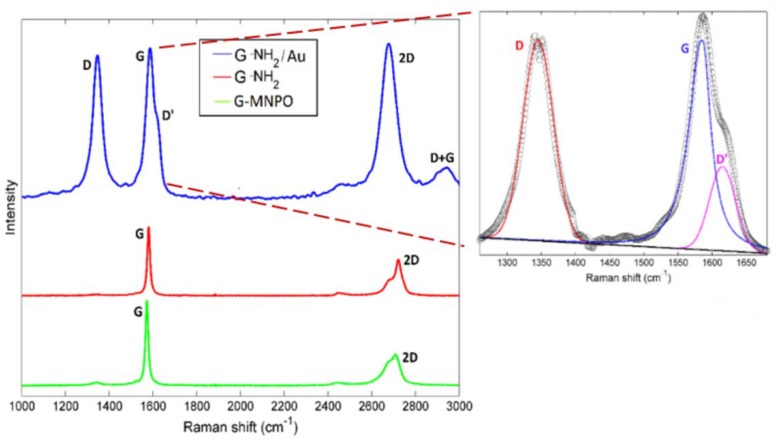
Raman spectra of G-MNPO, G-NH_2_, G-NH_2_/Au; the deconvolution of the D and G bands of the G-NH_2_/Au is reported in the inset.

In order to investigate the SERS enhancement of G-NH_2_/Au, Raman spectroscopy analysis was performed (Figure 7). The Raman spectrum of G-NH_2_ showed the G and 2D feature bands at 1580 and 2720 cm^−1^, respectively (Figure 7). The very weak D-peak was indicative of the high quality of G and the 2D band splitting indicated the presence of a multilayers system. All these Raman contributions were also evident in the G-NH_2_/Au nanocomposite, however some relevant differences were detected (Figure 7). Firstly, the increase of the intensity of all the peaks was observed, including a D band centered at about 1350 cm^−1^, as a result of a certain degree of disorder induced by Au NPs insertion within G layers. The strong electric field gradient induced by the metallic NPs determined an overall change of the dipole moment during the vibration, even in the absence of a polarizability change. On the other hand, when G and Au NPs were in close proximity, some Raman forbidden peak appeared, namely the D’ and the D+G contributions at about 1616 cm^−1^ and 2925 cm^−1^, respectively. These evidences can be determined by: (i) The insertion of Au NPs on G-NH_2_ platform, mainly at the edges of G layers (functionalized area of G layers) as suggested by computational studies [31] and (ii) reduced size of layers due to the mechanical effect of ultrasonication treatment adopted for the preparation of the nanocomposite. Moreover, the decrease of I_G_/I_2D_ ratio, from 1.58 to 0.98, pointed out a better exfoliation of G-NH_2_/Au with respect to G-NH_2_; the insertion of Au NPs between G-NH_2_ layers probably promoted their separation. Finally, the shifting of G and 2D bands suggested the anchorage of Au NPs on G surface. Raman signal was collected at several different sample locations to take into account the Au spatial homogeneity distribution within the nanoplatform. No significant changes were observed from one point to another one, which indicated that Au NPs were almost uniformly distributed within and/or on G layers.

### 3.3. SERS Properties of G-NH_2_/Au Platform

**Figure 8:**
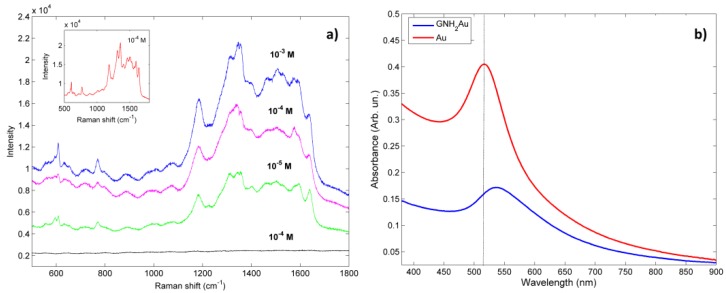
(**a**) SERS spectra of Rh6G (10^−5^, 10^−4^ and 10^−3^ M) onto G-NH_2_/Au platform; G-NH_2_ platform (10^−4^ M, black line) and AuNPs film (10^−4^ M Rh6G reported in the inset), by using the 638 nm laser excitation. (**b**) Optical absorption spectra of freshly prepared Au NPs (red line) and G-NH_2_/Au (blue line, 0.4 mg/mL).

In order to test the SERS properties, the platforms (G-NH_2_ and G-NH_2_/Au) were immersed for 30 min in Rh6G aqueous solutions at different concentrations (1 × 10^−3^, 2 × 10^−4^, 5 × 10^−5^ M) and then air dried. Raman spectra were acquired using two different excitation diode laser lines (532 nm and 638 nm). UV-vis spectroscopy was exploited to determine the appropriate laser wavelength for resonant excitation of the localized surface plasmon. In fact, SERS is more effective when incident radiation falling on the nanostructured substrate is completely absorbed by metal NPs, so that excitation of the localized surface plasmon can take place. The field enhancement is greatest when the plasmon frequency is in resonance with the incident radiation. In Figure 8b, the optical absorption spectra of the freshly prepared Au NPs and of G-NH_2_/Au were reported. Au NPs in water showed the characteristic Au surface plasmon resonance (SPR) band at 522 nm, due to the coherent oscillations of surface electrons interacting with an external electromagnetic field; whereas a red-shift (from 522 to 548 nm) and a decrease of the SPR intensity was observed in the G-NH_2_/Au sample, suggesting an increase of the spatial distance between each Au NPs and the others, due to their dispersion into each G foil and/or within the G layers. Moreover, a charge transfer from Au NPs to G occurred, resulting in a decrease in electron density, which, in turn, contributed to the red-shift and the intensity decrease of the SPR band. Moreover, it is well known that the coating of gold surface with graphene modifies the propagation constant of surface plasmon polariton (SPP), thereby changing the sensitivity to refractive index change and, in turn, the optical response of the entire system [36].

SERS spectra, acquired using the 638 nm laser excitation, showed the well-defined Raman Rh6G peaks at about 615, 777, 1189, 1314, 1366, 1513, and 1651 cm^−1^ (Figure 8a). The feature at 615 cm^−1^ was assigned to the C–C–C in-plane bending mode, the peak at 777 cm^−1^ to the C–H out-of-plane bending mode and the residual peaks to the aromatic stretching vibrations of C atoms. Raman features were clearly observable at 10^−3^ M concentration and less evident, but still visible, for lower Rh6G concentration values (down to 1 × 10^−5^ M). On the other hand, if the Rh6G aqueous solution was deposited onto a G-NH_2_ bare platform (i.e., without Au NPs), no Raman activity was detected even at a 10^−3^ M concentration. The Raman spectrum of G-NH_2_ was characterized only by broad asymmetric and low-intensity bands, at around 1580 cm^−1^ (referred as G band) and near 1330 cm^−1^ (referred as D band), typical of carbon-based materials. The Rh6G SERS spectra obtained on the G-NH_2_/Au platform were very similar to that obtained by using a substrate made from Au nanostructured film (Figure 8b). As a final remark, we observed that, by using a 532 nm laser excitation, no Raman signals could be collected in all the tested conditions. This unusual behavior can be explained taking into account the red-shifted observed SPR optical absorption, which certainly reduced the SERS effect. Summarizing, G layers positively influenced both the EM and the CM coupling, enhancing the SERS process due to the interesting optical properties, nanostructures high surface/volume ratio, and a great affinity between G and Au NPs.

**Figure 9:**
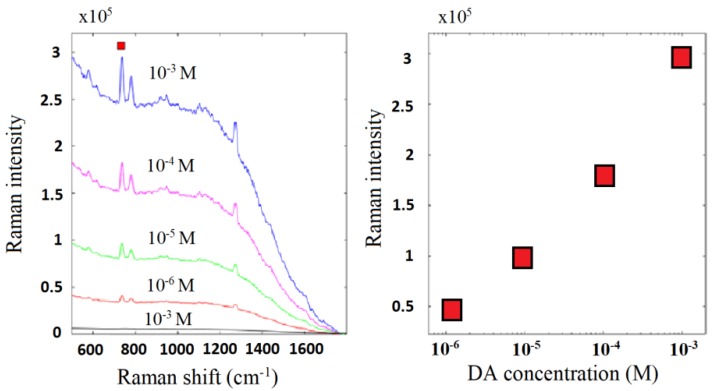
SERS spectra of DA tested at different concentrations (10^−6^, 10^−5^, 10^−4^, and 10^−3^ M) onto the G-NH_2_/Au platform along with the control test onto the G-NH_2_ platform (10^−3^ M, black line), on the left. The Raman intensity signal trend onto DA concentration is reported on the right. The excitation is the 638 nm laser line.

The ability of the G-NH_2_/Au nanocomposite to detect the biomolecules was tested using DA in a label-free configuration (Figure 9). DA is adsorbed on G surface through π–π stacking interactions [37]. The high surface area of G supporting the DA adsorption and diffusion processes was the primary condition for the efficient sensing of DA by SERS.

SERS spectra of DA showed several characteristic peaks centered at about 608, 767, 1349 cm^−1^. It is worth noting that other weak Raman features in the 1050–1300 cm^−1^ and 1500–1800 cm^−1^ regions can be detected despite the remarkable Raman background. The observed peaks were ascribed to the outside surface deformation of breathing, bending, and stretching vibrations of CH ring, bending vibration of NH, and aromatic C=C, respectively [38]. It is plausible that both EM and CM were involved in SERS signals, as already observed in reduced graphene oxide/silver nano-triangle sol substrate [39,40].

Au NPs between G layers behaved as “hot spots”, which allowed the detection of DA Raman signals, not observed in the G-NH_2_ bare platform. Since the distribution of Au NPs within G sheets played a fundamental role in determining SERS response and that it is known from the literature that one of the problems that still remain in question is the reproducibility and the repeatability of the spectra at low concentrations, we acquired SERS spectra in different points of the SERS substrate and at different times. We observed, point to point, very minimal variations in the intensity of some DA characteristic peaks without the degradation of nanocomposites.

## 4. Discussion

The results of this work has demonstrated the great potentiality for SERS applications of functionalized G obtained by covalent modification [41]. The cycloaddition protocol (i.e., 1,3-dipolar cycloaddition between mesoionic compounds and graphite) [31] furnished a G network decorated with ∆-1-pyrrolidine rings, mainly in the edge defected sites. This approach incorporated several interesting advantages: (i) Avoided the damage of sp^2^ G network; (ii) provided functionalized G with high degree of functionalization (i.e., 4.6%) and the NO_2_ group on pyrrolidine rings was reduced in good yield (XPS data indicated the reduction of almost half of the nitro groups in NH_2_ groups); (iii) the amine groups assisted the anchorage of Au NPs produced by PLAL on G surface; (iv) the dispersibility in water of the G-NH_2_ nanocomposite was enough for its deposition onto glass slide by aerography spraying technique. Au NPs included on G resulted in a hybrid nanocomposite (G-NH_2_/Au) that combined the stronger plasmonic-based EM of Au with the superior stability, adsorption, and quenching of G. This nanocomposite revealed strong interactions between the two entities. From its spectral features, the origin of these interactions could be attributed primarily to the strong electric field gradient induced by Au NPs that determined an overall change of the dipole moment during the vibration, even in the absence of a polarizability change. The SERS activity of the assembled Au NPs with the graphene platform is justified in terms of “hot spots”. Au NPs within the G-NH_2_ structure are “confined” to a certain region sensitive to the Raman scattering [42,43]; localization of light as surface plasmons in noble metal nanostructures enables their potential role in antennas, single molecule detection, and surface-enhanced Raman. Light localization by graphene structure induces the change of the electron structure of molecules due to their direct interaction with the surface in the first adsorbed layers. However, there is no “chemical enhancement” and the one in SERS is associated with very strong change of the electric field, when one moves away from the surface [44]. In this work, G-NH_2_/Au nanocomposite was used to identify the dye Rh6G and the neurotransmitter DA. DA is a catecholamine that plays a significant role in the functioning of central nervous, vascular, hormonal systems and its abnormal variation concentration in vivo has been linked to serious neurological diseases. The direct SERS quantification of DA in biological fluids remains a great challenge due to the low concentration (<10^−10^ M) and the high complexity of biological matrix. Raman features of Rh6G and DA were still detectable for concentration values down to 1 × 10^−5^ M and 1 × 10^−6^ M, respectively, although the sensibility of our system was found lower than the graphene-based SERS substrates reported in the literature for both analytes [24,45]. From our studies, it emerged that an improvement of G-NH_2_/Au sensibility is imperative before proposing it as substrate for the detection of DA in biological matrix. We hypothesized that the detection limit of G-NH_2_/Au nanocomposite could be improved by tuning the DA absorption properties on G and by setting the features, size, and shape of plasmonic noble metal NPs.

## 5. Conclusions

In summary, we investigated the SERS properties of a new graphene/gold nanocomposite (G-NH_2_/Au) obtained by combining Au NPs produced by PLAL technique with G covalently functionalized (G-NH_2_). After the chemical modification of G, the SERS platform was obtained by loading Au NPs on the G-NH_2_ surface and deposition of G-NH_2_/Au nanocomposite onto the glass slide by an aerography spraying technique. The chemical composition and the morphology of nanocomposites were investigated by micro-Raman XPS, STEM, and TGA analyses. STEM analyses showed transparent graphene sheets, with various dimensions, stacked onto each other, with a thickness of about 2–3 nm. Au NPs were detected as uniform spherical structures, with an average size of 15 nm, mainly distributed at the edges of the G layers. A good Au NPs loading was estimated by TGA and XPS analysis (i.e., 7.29% and 7.5%, respectively). This strategy allowed us to study SERS properties of G loaded with pure Au NPs without the influence of capping agents, surfactants, or salt produced in the chemical reduction of gold ions. SERS platform was tested to identify the dye Rh6G and the neurotransmitter DA; Raman features of Rh6G and DA are still detectable for concentration values down to 1 × 10^−5^ M and 1 × 10^−6^ M, respectively.

In conclusion, our platform possessed good stability and capability to reproduce the Raman signals without degradation although with low sensibility. Considering the feasibility of our method, further study will be devoted to improving the DA detection limits to refine the absorption properties of G-NH_2_ and the plasmonic effect of loaded noble metal NPs.

## Figures and Tables

**Table 1:** Atomic content percentage for G-MNPO, G-NH_2_, and G-NH_2_/Au samples as determined by XPS analysis and N 1s percentage determined by deconvolution of XPS N 1s band. Weight content percentage of G-NH_2_/Au calculated by XPS values (at the bottom).

Atomic Content Percentage Determined by XPS Analysis					N 1s Content Percentage Determined by Deconvolution of XPS N 1s Band	
Sample	Au	C	O	N	N 1s (N=C, NH_3_^+^)	N 1s (NO_2_)
G-MNPO	-	74.8	19.0	6.2	58.82	41.18
G-NH_2_	-	89.3	7.4	3.3	79.95	20.05
G-NH_2_/Au	0.5	89.0	9.4	1.1	83.66	16.34
Weight content percentage calculated by XPS values						
G-NH_2_/Au	7.5	80.2	11.3	1.2		
